# Gender Effect on the Relationship between Talent Identification Tests and Later World Triathlon Series Performance

**DOI:** 10.3390/sports9120164

**Published:** 2021-12-06

**Authors:** Alba Cuba-Dorado, Veronica Vleck, Tania Álvarez-Yates, Oscar Garcia-Garcia

**Affiliations:** 1 Laboratory of Sport Performance, Physical Condition and Wellness, Faculty of Education and Sport Sciences, University of Vigo, Campus A Xunqueira s/n, 36005 Pontevedra, Spain; tanalvarez@uvigo.es (T.Á.-Y.); oscargarcia@uvigo.es (O.G.-G.); 2 Centre for the Interdisciplinary Study of Human Performance (CIPER), Faculdade de Motricidade Humana, University of Lisbon, Estrada da Costa, Cruz Quebrada-Dafundo, 1499-002 Lisbon, Portugal; vvleck@fmh.ulisboa.pt

**Keywords:** elite, testing, prediction, triathlete, talent, gender

## Abstract

Background: We examined the explanatory power of the Spanish triathlon talent identification (TID) tests for later World Triathlon Series (WTS)-level racing performance as a function of gender. Methods: Youth TID (100 m and 1000 m swimming and 400 m and 1000 m running) test performance times for when they were 14–19 years old, and WTS performance data up to the end of 2017, were obtained for 29 female and 24 male “successful” Spanish triathletes. The relationships between the athletes’ test performances and their later best WTS ranking positions and performance times were modeled using multiple linear regression. Results: The swimming and running TID test data had greater explanatory power for best WTS ranking in the females and for best WTS position in the males (R^2^a = 0.34 and 0.37, respectively, *p* ≤ 0.009). The swimming TID times were better related to later race performance than were the running TID times. The predictive power of the TID tests for WTS performance was, however, low, irrespective of exercise mode and athlete gender. Conclusions: These results confirm that triathlon TID tests should not be based solely on swimming and running performance. Moreover, the predictive value of the individual tests within the Spanish TID battery is gender specific.

## 1. Introduction

Triathlons involve sequential swimming, cycling and running. Only athletes with around a top 150 world ranking may compete in the World Triathlon Series (WTS), i.e., the highest level of competition below the Olympic Games. The annual WTS circuit involves up to nine races over the Olympic (OD) (1.5 km swim, 40 km bike, 10 km run) and Sprint (0.75 km swim, 20 km bike and 5 km run) distances, plus a (more highly scored) Grand Final over the OD. The final WTS season ranking equates to a world championship ranking. Climatic conditions permitting, at any given event, both sexes compete over the same race distances. Within-competition analyses [1,2,3] have, however, demonstrated that gender differences exist in the relative importance of individual swimming, cycling and running performance to the overall race result in elite triathlon.

The running discipline makes the most decisive contribution to finishing position in World Cups [3,4], World Championship/WTS Grand Finals and the Olympic Games, but more so for males. Swimming performance affects final race position less [1,3]. Vleck et al. [4] reported World Cup performance to relate both to average swimming speed and position at the swim exit. Slower swimmers must reduce their time gap to the leading bike pack(s) by the run start. Swimming speed more strongly affects finishing position in females, however [1,3]. Because of their differences in speed and performance density, cycling performance can become more important in females [2]. Females form more, smaller, cycle packs and are likely less able to bridge gaps between such packs [3].

Pacing strategy also affects elite performance. In both genders, speed up to the first buoy of a one-lap World Cup swim was associated with finishing position. The top 50% of males swam this faster than the bottom 50%. Thereafter, swimming speeds were similar [3]. Elites also reportedly adopt a positive or a reverse J-shaped pacing strategy out of the bike–run transition (T2), running faster within the first kilometer and, when faced with direct opponents, over the final 400 m or less of the run [3,4,5,6]. Because they generally exit T2 in larger groups, and have similar 10 km times, this ability to do an “end spurt” may prove to be especially important in males.

To date, only one race analysis [2] has exclusively focused on WTS events. It examined the relative influence of the three triathlon disciplines on WTS performance, across two Olympic cycles, in 1670 males and 1706 females. Competitors were grouped by finishing position (G1: 1st–3rd place; G2: 4th–8th place; G3: 9th–16th place and G4: ≥17th place). The main effects of years and rank groups were compared. For females, swim and bike segment differences existed only between G4 and the other groups (*p* = 0.001–0.029). Each group differed from the other for the run (*p* < 0.001). For males, swimming performance differed only between G4 and the other groups (*p* = 0.001–0.039). Although running was where differences existed between all the groups (*p* < 0.001), it was apparently important for success that a good runner be positioned with the first cycling pack. Bike splits did not differ, however, between the different male groups, for whom “the bike leg seemed to be a smooth transition towards running” [2]. Only the first 16 women had similar bike splits, however. Even at the WTS level, females likely divide up into more bike groups, further apart, and may therefore be more affected by residual fatigue at the run start than males [2].

These gender differences in the relative extent to which performance in its component disciplines influences triathlon performance [2,3] may have important consequences for talent identification (ID). The extent to which talent ID test results relate to adult performance is used to justify the resources that are allocated to it. This then can markedly impact the selected athletes’ likelihood of sporting success. To date, however, most triathlon talent ID research has either involved mixed-gender groups or just males. “While … we know very little about predictors of talent in elite sport, we know even less about predicting talent in female athletes. Given the often-unique development systems for high-performance female athletes, this discrepancy might limit our ability to gain a deeper understanding of talent, and … lead to potentially harmful consequences for the female athlete population” [7].

We do not know the extent to which the individual predictive capacities of the tests within the Spanish Triathlon Federation (FETRI) battery differ with gender. Although they were previously reported to have little predictive capacity [8] (in that case for National Championship-level performance), the study sample was of mixed gender. The extent to which FETRI talent ID test results separately relate to male and female WTS performance is unknown. Therefore, the aim of this study was to explore, as a function of gender, the extent to which the FETRI talent ID test results predicted the later WTS performance of Spanish triathletes.

## 2. Materials and Methods

### 2.1. Study Design

An explanatory transverse study design was used to establish the relationship between the FETRI talent ID test results (Table 1), and later WTS performance, of both males and females (Table 2). Two independent analyses were carried out: (1) for best end-season WTS ranking (RankWTS) and (2) for best WTS individual event position (PositionWTS).

### 2.2. Participants

Our subjects were considered “the successful products” of the FETRI talent ID process because they either obtained a final WTS ranking or raced at the WTS level in 2009–2017.

### 2.3. Procedures

From 2009 to 2016, 3502 fourteen to nineteen year-olds underwent the FETRI test battery. This comprised two freestyle swimming tests (i.e., S100: a 100 m time trial; S1000: a 1000 m time trial) in a 25 m pool, and two running track tests (i.e., R400: a 400 m time trial; R1000: a 1000 m time trial). Each test performance time (in seconds, T) was scored, up to a maximum of 12 points, using a proprietary FETRI age- and sex-specific scale (Table 1) [8,9,10]. Those who scored 8 or more points in each of at least three tests were then separated into age- and gender-specific subgroups. One-year age groups, as opposed to category (e.g., “junior” or “cadet”) groupings, were used to offset relative age effect(s) [11]. Each individual’s total test performance time was then expressed as a percentage of the fastest ever summated four (swim and run) test times for their age subgroup (variable 4R).

The World Triathlon results database (see www.triathlon.org accessed on 1 april 2021) was then used to identify the “successful” 24 males and 29 females to which this study pertains before their talent ID data were obtained from FETRI.

The research protocol was both in accordance with the Declaration of Helsinki and approved by the local University Ethics committee.

### 2.4. Statistical Analysis

Sufficient sample size was calculated using G * Power v3.1.9.4 for Windows (Heinrich-Heine-Universität Düsseldorf, GER), resulting in an N of 48 being considered appropriate (effect size = 0.36; α error probability = 0.05; power = 0.95). Sample normality, linearity and homoscedasticity were assumed after carrying out the Kolmogorov–Smirnov test. Pearson’s bivariate correlation coefficient was used to determine the inter-relationships between test times. The relationships between the successful athletes’ talent ID test results and their RankWTS and PositionWTS data were modeled using step-by-step multiple linear regression. The degree of data independence was calculated using the Durbin–Watson test (and assuming independence of values between 1.5 and 2.5). Variance inflation factor (VIF) values above 10 were taken to indicate multicollinearity. The 95% confidence level was considered statistically significant. All the analyses were performed with the Statistics Package for the Social Sciences (SPSS version 19.0 for Windows, SPSS Inc., Chicago, IL, USA).

## 3. Results

The athletes’ swimming and running test times (Table 3) were positively intercorrelated in the males. The correlation coefficients were large for between S100 and S1000 (r = 0.853, *p* = 0.001), and moderate for between S100 and R400 (r = 0.431, *p* = 0.045), S100 and R1000 (r = 0.431, *p* = 0.045), S1000 and R1000 (r = 0.552, *p* = 0.006) and R1000 and R400 (r = 0.742, *p* = 0.001). In the females, only R400 and R1000 (r = 0.836, *p* = 0.001), and S100 and S1000 (r = 0.750, *p* = 0.001) were significantly intercorrelated.

Table 3 also presents the linear regression models for the relationships between talent ID test results and both RankWTS and PositionWTS. For the values obtained with the Durbin–Watson test (with the exception of “all cases” in the response to RankWTS and “females” in the response to PositionWTS) independence of the residuals was assumed. As no VIF values exceeded 3.5, multicollinearity was not considered to be a problem.

The females’ talent ID test results best explained their best WTS ranking. According to the value of the adjusted coefficient of determination (R^2^_a_) (*p* ≤ 0.007), 33.6% of the total variance in best female ranking at the end of the season was explained by 4R_R1000_, T_S100_ and 4R_S1000_. The regression equation was:

Female RankWTS = −960.306 + 7.060 × 4R_S1000_ − 2.852 × 4R_R1000_ + 10.285 × T_S100_ (1)

The males’ talent ID test results, however, better explained best individual WTS race position than best male season-end WTS rankings. The corresponding R^2^_a_ (*p* ≤ 0.009) indicated that 37.2% of the total variance in male PositionWTS was explained by S100 and S1000 performance times:

Male PositionWTS = −101.692 − 0.315 × T_S1000_ + 5.933 × T_S100_ (2)

## 4. Discussion

Few data relating to the accuracy of early talent decisions exist [7]. “High-quality scientific research is needed in order to (a) determine the reliability and validity of talent identification and selection initiatives, (b) inform evidence-based models of athlete development, and (c) identify gaps in current understanding and directions for future work. Ineffective or inaccurate decisions have important repercussions for all stakeholders involved (e.g., dropout, decreased motivation, misplaced resources, and investment)” [12].

Baker et al. [12] stated that it is “imperative to better understand factors related to female-specific talent development.” Although their review of the talent-related literature indicated over thirty such triathlon studies to have taken place thus far, we believe this to be the first one to examine the accuracy of talent decisions for expert male and female triathletes. FETRI test performance poorly predicted WTS performance in both genders. In our “successful” females, talent ID results explained 33.6% of the variance in best end-season WTS ranking (i.e., the more important of the two variables) and 26.8% of the variance in best individual WTS race placing. In “successful” males, the corresponding values were 32.6% and 37.2%. In our results, when both genders were analyzed together (Table 3), the explanatory power of the tests dropped (from 33.65% in females and 32.6% in males) to 30.3% overall for best end-season WTS ranking and to 36.5% for best individual WTS event position. This is both unsurprising, given that the constraints and developmental models of females differ from those of males, and confirms that the predictive capacity of the battery FETRI talent ID test is gender specific.

The explanatory power of the *individual* FETRI tests for best WTS performance also differed with gender. Again, this finding, given the gender differences in the relative importance of performance within each triathlon discipline that exists at the WTS level, was expected, since the “disciplines that precede the triathlon run appear to have more impact on overall race performance in females than they do in males. In males, where the performance density is better, the ability to complete a fast, sprint type, run finish can be definitive” [2].

However, we did not set out to predict WTS performance *per se.* Rather, we explored how much of the variance in male and female WTS performance could be explained by performance in each of the FETRI swim and run tests. The prognostic validity of these predictors for draft-legal OD triathlon performance is unconfirmed, nor are the optimal pacing strategies within the WTS competition yet known. However, the (44 race) analysis that was conducted by Piacentini et al. [2] found differences in swim times, bike times and run times between podium (G1), 4th and 8th place (G2), 9th and 16th place (G3), and ≥17th place (G4) female WTS finishers. Within males, these differences occurred only for swimming and running. No difference in swimming segment times was noted, in both sexes, between the first three such groups. It was clearly important to overall WTS performance that good runners were able to position themselves within the first cycling packs to reach T2.

Piacentini’s study population would have included our males and females, classing them as G1–G4 and G4 triathletes, respectively. In males, therefore, we expected to see significant relationships between the swim and run FETRI test results and performance. Piacentini et al. [2] observed that for males, exiting the water and exiting T2 close to the leader, with a fast running split, appeared to be major determinants of success. In females, both the T1 and T2 exits were important, as was a very fast run split. In males, G1 also differed from G2 to G4 as regards entry into T1 and exit from T2. Entry into T1 was less important than exit from T2, and run sprinting ability was likely more important, in males. In females, position out of T2 was likely more important for overall performance than was sprinting ability.

The 1000 m swim test featured in all our models, as did the 100 m swim test, in all cases apart from the males’ best RankWTS. As regards the running tests, when both genders were analyzed together, the 1000 m featured in both the RankWTS and the PositionWTS models. When we analyzed each sex separately, the 1000 m run only had predictive power for the females’ best RankWTS. In males, the 400 m run test predicted best WTS finishing place. Again, our results broadly agree with Piacentini et al. [2]. The triathlon run is a more decisive contributor than the swim and the bike to the race result, at multiple levels of elite competition. Observed correlation coefficients between triathlon swim and run performance, and overall finishing position, of −0.36–0.42 vs. 0.88–0.94 for males and −0.47–0.49 vs. r = 0.71–0.85 for females, respectively, support this [1,2,3]. However, the FETRI swim tests explained more variance in WTS performance than the run tests did. Some possible reasons why this was the case, aside from the relative heterogeneity that existed in the performance levels of our two gender groups, are detailed below.

The S100 and S1000 tests that our triathletes underwent in the FETRI talent ID battery are thought to be largely anaerobic and aerobic, respectively. To *some* extent, they reflect the reality of elite competition, “for which the ability to start fast … and then maintain a steady swim pace below 90% of … maximal speed could be seen as a preferred pacing strategy” [6]. World-Cup-level triathletes have been noted to swim faster up to the first swim buoy and then to sustain a relatively slower pace over the rest of (each) swim lap [3,4]. This first buoy is normally *250–350 m* from the shore/pontoon. How fast the speeds over the first 100 m, compared to the rest, of that 250–350 m are is unknown, however. No “surge” data are available for these intermediate sections, nor do we know to what extent elites speed up just before the swim–land transition(s). Yet, it seems appropriate that the talent ID test battery includes both a short and a longer swim. However, we do not know which of the various test distances used by different federations (e.g., the 200 m and 400 m of Italy that Bottoni et al. [13] reported vs. the 100 m and 1000 m of Spain) is the optimal combination.

Perhaps, however, the potential relevance of the shorter swim test is increasing over time. The 2017/2018 WTS season included more sprint distance events [6]. The best WTS triathletes from a given country are fairly likely to also represent it at the Olympic Games, not only over in the OD but also within the mixed team relay (MTAR). MTAR competitors do a 300 m swim, a 6.6 km cycle and *a 1 km* run before handing over to a teammate, in the given order of female–male–female–male. Sharma and Periard [5] reported that Australian athletes competing at the 2014 MTAR World Championships also implemented positive swim pacing, “likely due to a desire to be at the head of the swim group and avoid being disrupted (i.e., stroke mechanics, breathing, “fighting” for position) by swimming in a large group.” It makes sense that the ability to make a fast start at the very onset of the swim may become increasingly important in elites, although insufficient data are yet published relating to this point.

As for running, the 400 m and 1000 m run tests that our triathletes performed as part of the FETRI talent ID battery have a marked anaerobic component. Although the triathlon run was traditionally thought to be predominantly aerobic, the situation in elite draft-legal races is more nuanced [5]. This may partly be because of the common athlete tactic, which for the OD seems to contradict physiological principles, of a fast start out of T2. Within-race analyses usually report this fast start to occur over the first *1000 m*, i.e., over the same distance as the longer FETRI run test, but researchers traditionally position their cameras at 1 km from T2. Etxebarria et al. [14] also examined (only male) pacing over 2.5 km sections of the 10 km run after 2016. They did this over 14 World Cup and WTS events, over three years and 726 race outcomes. The 171 males ran “the first lap of the standard four lap circuit substantially faster than laps 2 (~7%), 3 (~9%) and 4 (~12%).”

We are unaware of any speed data relating to between when an athlete racks their bike and perhaps trying to avoid “getting stuck in a traffic jam” [15]) when he/she gets out on to earlier parts of the run. However, “at the speeds run by elite male triathletes drafting may have some benefit on oxygen consumption and therefore performance; as such triathletes may want to adopt a faster start to keep up with leading runners. Additionally, triathletes present in the front group and thus in contention for the victory could have a psychological advantage over chasing athletes and therefore perform better…” However, balancing the benefits of drafting against the physiological cost of a faster start would be a key consideration, with the potential for specific athletes (i.e., those with “… higher anaerobic tolerance qualities to target an aggressive pacing strategy”) [5].

The athletes, particularly males, as, historically, they have greater performance density, who can do this *and* then do a “kick” or spurt over the last *400 m or less* of the 10 km, *when/if needed,* are likely to possess a competitive advantage over those who cannot. Interestingly, Sharma and Periard [5] demonstrated that fast run starts also occur within the MTAR. It is unknown to what extent they occur in Sprint distance WTS races, but these can account for approximately a quarter of WTS events [2]. Such information is therefore relevant to future talent ID and development.

We expected our low predictive power of the FETRI data for WTS performance. Talent ID tests generally possess low predictive power [7,16]. Moreover, Cuba-Dorado et al. [8] already found this, albeit in a mixed-gender sample, in relation to the draft-legal Spanish National OD Championships performance of the same year. Piacentini et al. [2] already demonstrated smaller time differences to exist between groups and athletes at the WTS level than have been previously recorded for lower-tier elite events. Obviously, differentiating between individuals who are at the same level of participation is particularly challenging at the top level [12]. The low predictive power shown by our results notwithstanding, it is heartening that the FETRI test battery predicted better WTS rankings than it did best individual WTS event placings. Rankings are both a measure of performance consistency and (partly) circumvent the problem of triathlon not having standardized course lengths or course difficulty ratings. The WTS ranking is by far the more important of the two variables.

We note that the predictive power of the talent ID tests for the WTS ranking was slightly better in females. A clear relative age effect (RAE), even for one-year groupings, was reported in males on this *same* test battery. It is less evident in females, which may partly explain the gender difference [11]. Despite the fact that an RAE was demonstrated for the male triathletes who competed in the 2012 Olympic Games [17], none of the investigations of the Spanish test set to date (including this one) seem to have adequately accounted for the physical maturity levels of the individual athletes at the time of its administration. Basing the 4R data around one-year athlete groups, as opposed to the two-year performance categories that exist in Spain, only does so to some extent. At least in males, the RAE has been demonstrated even within these one-year age categories [11]. This study did not account for the time interval between the athletes doing the talent ID tests and their WTS results, although Cuba-Dorado et al. [8] also reported that the length of the time interval between the administration of the Spanish talent ID tests and the competition under investigation affected the explanatory power of the tests for said competition results. Deliberate sports practice over a long period of time has certainly been shown to influence the difference in performance between experts and novices. This is especially so for running [18].

However, given *how* low the explanatory power of the FETRI battery is (and the advent of the MTAR as an Olympic event), in both genders, it may be worthwhile re-evaluating how it is made up. Our results confirm, for the WTS, what Bottoni et al. [13] wrote a decade ago, i.e., that triathlon talent ID test batteries should not exclusively focus on evaluating swimming and running performance. Bottoni et al. [13] made that assertion on the basis of comparative retrospective data, dating back to when their subjects were 14 years of age, for sixty-six top 5 male World Cup, World Championship and Olympic Games finishers over the period 2000–2008 vs. top 15 Italian males. The data indicated that cognitive/psychological assessment [13,19], and assessing the athlete’s level of physiological and psychological maturity [20] are good ideas. So too is the collection of data on the athlete’s previous performance/training history [7].

We emphasize that talent identification should focus on recognizing athletes who have the *potential* for future excellence [21], because early entry into the talent ID system and its associated benefits can be a crucial factor in talent development [22]. It remains important that the scoring criteria for identifying athletes via the existing FETRI tests are not too restrictive. This would allow enough triathletes to continue within the talent identification program and, later on, be assessed both on their rates of improvement [13] and more specific aspects of elite performance such as their technical and tactical skills [22].

In addition to being accused of relying “on a relatively small number of heavily weighted variables measured in isolation from the sport context,” many talent ID batteries stand accused of “adopting testing batteries that do not accurately represent the sport demands” [16]. Johnston and Baker’s comment is definitely pertinent to the sport of triathlon, which “*must be seen to be more than the sum of the sports of which it is made up*” [23]. Using the 4R variable, which partly accounts for performance across shorter and longer distances of each of two of the three triathlon disciplines, measured separately, is not enough to do this. A key reason for this likely is that the triathlete’s cycling ability, which can to some extent compensate for a poor position, relative to the leading bike packs, at the swim exit, and affect how fresh they are at the run start [24], is ignored.

It is logical that once an athlete has been selected via the initial talent ID process, a more detailed assessment of their potential to achieve elite-level performance be conducted [5,6]. Said assessment should include measurement of the universally acknowledged key triathlete ability of starting each discipline with minimal residual fatigue from the preceding disciplines. This could be done by a cycle–run-specific transition test, at least three lab-based versions and two field-based versions of which exist [25]. The five tests all assess the extent to which the triathlon run start is influenced by residual fatigue from the bike section. They perhaps vary in their suitability of application for different scenarios and different athlete groups.

We draw the reader’s attention to the fact that both laboratory and anecdotal race data suggest that the athletes who are less well adjusted at the run start exhibit decreased stride lengths. “Athletes with longer running contact times may produce the same impulse for a lower metabolic cost than their stiff … and/or fast twitch counterparts” [26]. It may be that the athletes who exhibit a higher stride frequency at the run start *may then pay for this by not subsequently being able to exert a “kick” or speed spurt, if it is needed, at the run finish*. The extent to which the athlete exhibits a less efficient running style, and their pacing over the rest of the race, may be related to their cycle: anaerobic power reserve (APR) [27] and/or run-specific anaerobic speed reserve (ASR) [28]. These are typically defined as the difference between maximal sprint power output and power output at maximum oxygen uptake (VO_2_max) and the difference between maximal sprinting speed and running speed at VO_2_max, respectively. We note that, although the study had methodological issues, when the performances of senior and junior males on the Spanish bike–run field test (i.e., a 30 min steady-state cycle plus a self-paced 3 km run) [29] were compared, they were found to differ in run pacing style. Because having a good APR/ASR may differentiate between athletes who are relatively homogenous as regards other, commonly measured, markers, and because APR/ASR development relates to both developmental and training levels, it may be a useful longitudinal marker. Cycle and run field tests for it exist [27,30]. The ability to recover from cycle surges will of course affect the athlete’s fatigue at the run start. This may then compound the negative effects of positive run pacing. Since “a lower anaerobic capacity leads to an inability to accelerate at the end of the race, which can accrue because of a reliance on anaerobic energy to maintain pace in an athlete of inferior running economy” [28], the higher the athlete level, the more relevant APR/ASR testing may become.

“Parameters which differentiate athletes at one competition level may not be as valuable as athletes’ progress” [20]. Variability in the extent to which the performance in the same component of the test battery contributes to successful performance across different levels of competition is also something to explore. It would be interesting to study, on a longitudinal basis, whether monitoring the factors that are perhaps then identified as distinguishing “expertise” from “eminence” (e.g., between being able to achieve a top 50 placing at National Triathlon Championships vs. obtaining a high enough world ranking to be competing at the WTS level) [16] could be used to distinguish between triathletes with differing capabilities of sustaining a given pacing strategy, and then to train this capacity [19].

Given all of the above, it can be considered both a strength and a limitation of our study that it focused specifically on the extent to which the *Spanish* talent ID test battery explained later WTS-level performance. It is only because Spain has been one of the top-performing countries in the world for triathlon that we were able to achieve such high numbers of “successful” elites, of both genders, for our regression analysis. Had we been able, however, to extend this analysis to involve the “successful” athletes from other countries as well, we would probably have been able to gain better insight into exactly which of the different talent ID test distance combinations that are implemented by such countries best explains the variance in top-level triathlon performance.

## 5. Conclusions

We confirmed, at the WTS level, the finding that “retrospective analysis of running and swimming performance outcomes *only* is not an appropriate method for predicting future triathlon success” [13]. However, as research into the efficacy of talent selection decisions, particularly that which compares the two sexes, is rare [7,19], our demonstration of clear gender differences in the explanatory power of the individual tests within the talent ID battery for WTS performance is noteworthy. So too are the clear corollaries between these gender differences and how males and females were actually performing in the WTS-level competition.

## Figures and Tables

**Table 1:** FETRI scoring system for performance of the individual components of the Spanish triathlon ID test battery.

Age	Points	Females				Males			
		R400	R1000	S100	S100	R400	R1000	S100	S1000
14 years	3	1:19.0	3:55.0	1:17.0	14:10.0	1:07.0	3:14.0	1:09.0	13:30.0
	10	1:12.0	3:20.0	1:10.0	13:00.0	1:00.0	2:46.0	1:02.0	12:20.0
	12	1:10.0	3:10.0	1:08.0	12:40.0	0:58.0	2:40.0	1:00.0	12:00.0
16–17 years	2	1:19.0	3:55.0	1:17.0	14:10.0	1:07.0	3:14.0	1:09.0	13:30.0
	10	1:11.0	3:15.0	1:09.0	12:50.0	0:59.0	2:43.0	1:01.0	12:10.0
	11	1:10.0	3:10.0	1:08.0	12:40.0	0:58.0	2:40.0	1:00.0	12:00.0
18–19 years	1	1:19.0	3:55.0	1:17.0	14:10.0	1:07.0	3:14.0	1:09.0	13:30.0
	10	1:10.0	3:10.0	1:08.0	12:40.0	0:58.0	2:40.0	1:00.0	12:00.0

Athlete’s age was determined by their age on 31 December in the year of the tests. Performance times are given in mm:ss.s. S100: 100 m freestyle swimming test, S1000: 1000 m freestyle swimming test. R400: 400 m running test; R1000: 1000 m running test.

**Table 2:** Performance times achieved within the FETRI talent identification test battery; best seasonal WTS rankings, best seasonal finishing positions within an individual WTS event achieved by “successful” Spanish triathletes (mean ± SD).

Talent ID Test Performances	Females			Males		
	*N*	Time (min:ss.s ± s)	4R (%)	*N*	Time (min:ss.s ± s)	4R (%)
S100	29	01:05.82 ± 2.56	93.28 ± 4.15	24	01:00.81 ± 3.52	90.04 ± 5.30
S1000	29	12:53.07± 42.01	89.64 ± 16.46	24	12:27.38 ± 55.54	89.38 ± 6.71
R400	28	01:10.96 ± 3.99	88.78 ± 7.41	22	00:58.51 ± 2.92	92.95 ± 4.27
R1000	29	03:17.72 ± 9.64	93.90 ± 5.80	23	02:46.26 ± 9.98	93.57 ± 4.23
WTS Results	Females			Males		
	*N*	Mean ± SD	Min-Max	*N*	Mean ± SD	Min-Max
Rank	26	100 ± 44	44–159	24	83 ± 53	1–165
Position	21	36 ± 14	18–58	19	24 ± 18	1–55

*N*: number of triathletes; F: females, M: males, 4R: percentage of the best ever times within the talent identification test, S100: 100 m freestyle swimming test, S1000: 1000 m freestyle swimming test. R400: 400 m running test; R1000; 1000 m running test. WTS: World Triathlon Series; Rank: best ranking position obtained by the triathletes within the WTS (i.e., RankWTS), Position: best position obtained by the triathlete within an individual WTS race (i.e., PositionWTS).

**Table 3:** Summary of the linear regression models for the best seasonal WTS ranking position and best seasonal finishing positions within an individual WTS event achieved by “successful” Spanish triathletes.

WTS Performance Predictors								
		Predictors	R^2^	R^2^_a_	R	Error	Sig	D-W
Rank	All	4R_R1000_, T_S100_, 4R_S1000_	0.346	0.303	0.588	40.710	0.000	1.16
	F	4R_R1000_, TS100, 4R_S1000_	0.415	0.336	0.645	35.479	0.007	1.73
	M	T_R400_, 4R_S1000_	0.391	0.326	0.625	44.863	0.009	1.56
Position	All	4R_R1000_, T_S100_, T_S1000_	0.415	0.365	0.644	13.577	0.000	2.10
	F	T_S1000_, 4R_S100_	0.342	0.268	0.584	11.977	0.023	1.13
	M	T_S1000_, T_S100_	0.442	0.372	0.664	14.351	0.009	1.69

R: Multiple linear regression; R^2^: R Square; R^2^_a_: adjusted R2; Error: standard error; D-W: Durbin–Watson test. F: females, M: males, 4R: percentage of the best ever times within the talent identification test, S100: 100 m freestyle swimming test, S1000: 1000 m freestyle swimming test. R400: 400 m running test; R1000; 1000 m running test. WTS: World Triathlon Series; Rank: best ranking position obtained by the triathletes within the WTS (i.e., RankWTS), Position: best position obtained by the triathlete within an individual WTS race (i.e., PositionWTS).

## Data Availability

Data of the talent ID test battery and the National Sports Development Program scoring scales were obtained from the FETRI website (see www.triatlon.org accessed on 1 April 2021). World Triathlon Series performance was extracted from the World Triathlon results database (see www.triathlon.org accessed on 1 April 2021).
